# Obesity‑related kidney disease: a review on ultrasound applications

**DOI:** 10.1186/s13052-025-02090-9

**Published:** 2025-08-12

**Authors:** Armando Di Ludovico, Costanza Pucci, Giovanni Grassi, Gian Luca Chabert, Giuseppe Ledda, Ilaria Mascioli, Saverio La Bella, Francesca Ciarelli, Luciana Breda, Cosimo Giannini, Angelika Mohn, Francesco Chiarelli, Antonio Corsello

**Affiliations:** 1 Department of Pediatrics, University of Chieti“G. D’Annunzio”, Chieti, Italy; 2 Department of Clinical Sciences and Community Health, University of Milan, Milan, Italy; 3 Department of Radiology, Azienda Ospedaliero Universitaria (A.O.U.) di Cagliari – Polo di Monserrato, Cagliari, Italy; 4 UOC Rheumatology and Autoinflammatory Diseases, IRCCS Istituto Giannina Gaslini, Genoa, Italy; 5 Department of Clinical-Surgical, Diagnostic and Pediatric Sciences, University of Pavia, Pavia, Italy

**Keywords:** Pediatric ultrasound, Obesity, Kidney, Nephrology, Metabolic syndrome, Doppler

## Abstract

Pediatric obesity is a growing global health concern, associated with metabolic, cardiovascular, and kidney complications. Early identification and intervention are crucial to preventing long-term morbidity. This review examines the epidemiology, pathophysiology, and clinical implications of childhood obesity, focusing on its impact on kidney health. We discuss non-invasive diagnostic tools, including kidney ultrasound, and evidence-based management strategies. Obesity in children is linked to hypertension, insulin resistance, and early signs of kidney dysfunction, including increased kidney echogenicity and hyperfiltration. Ultrasound findings may serve as early markers of kidney involvement, potentially guiding risk stratification and intervention. Addressing pediatric obesity requires a multidisciplinary approach, incorporating lifestyle modifications, medical management, and early detection of organ damage. The aim of this narrative review is to summarize the pathophysiology of pediatric obesity-related kidney damage, current evidence on ultrasound‐based screening, and possible diagnostic techniques. Further research is needed to validate ultrasound as a routine screening tool for obesity-related kidney disease in children.

## Introduction

The World Health Organization defines severe obesity as + 3 Z-scores relative to the median growth reference curves [1]. Child obesity is a worldwide epidemic, and the prevalence of severe and clinically relevant obesity in children has been steadily increasing, ranging from 1% of the population in Northern Europe to 5.5% in Southern Europe in 2019, or even higher percentages in the United States [2]. The impact of obesity on kidney structure and function is well-established in adults, resulting from both direct glomerular injury and comorbidities such as diabetes and hypertension [1, 3]. There is evidence to suggest that overweight and obesity are associated with adverse effects on kidney function in children as well [4]. The need for timely, non-invasive diagnostic strategies is especially critical to enable early diagnosis and tailored management, aimed at slowing down the progression of kidney disease.

The first-line diagnostic imaging in this context is ultrasonography/ultrasounds (US), a non-invasive and accurate technique fundamental in detecting early kidney changes, especially in obese pediatric patients. Its various applications play a pivotal role in the comprehensive assessment of obesity-related kidney damage. High-resolution US provides quantitative morphologic and hemodynamic parameters such as cortical thickness, parenchymal volumetric analysis [5], and kidney arterial resistive index (RI), which may be useful in the diagnosis of obesity-related hypertension in conditions like nephrotic syndrome [6]. According to the American Academy of Pediatrics (AAP) guidelines on high blood pressure screening, kidney US may be used as a noninvasive screening study for the evaluation of possible renal artery stenosis in normal-weight children and adolescents ≥ 8 years of age who are suspected of having renovascular hypertension (moderate strength), and they consider kidney US as a valuable screening test in all patients < 6 years of age or those with abnormal urinalysis or kidney function [7]. Moreover, US represents a crucial element in guiding percutaneous kidney biopsy, providing thus histologic diagnosis and further treatment [8], and being a valuable tool in diagnosing obesity-associated kidney changes and preventing complications [9]. According to AAP guidelines, kidney and bladder US is recommended after a first febrile urinary tract infection in children aged 2–24 months, with voiding cystourethrography reserved for cases where US reveal abnormalities [10, 11]. However, US alone (not contrast-enhanced) has shown poor sensitivity for detecting genitourinary anomalies and may miss significant cases of kidney damage, as evidenced by scintigraphy [12]. On the other side, contrast-enhanced US (CEUS) is an approved application in pediatric patients for the evaluation of vesicoureteral reflux, with adequate sensitivity [13]. Additionally, pediatric hypertension guidelines advise Doppler US for suspected renovascular hypertension in normal-weight children ≥ 8 years old, whereas technical limitations make its use challenging in obese children [14].

These approved screening indications should be clearly distinguished from the broader diagnostic uses of US in pediatric nephrology, such as evaluating kidney morphology, cortical thickness, and volume in obese children.

This review aims to explore the mechanisms of kidney damage secondary to obesity in children and to highlight how US stands out as a tool for early diagnosis and interventions.

## A combined pathophysiology

Obese children face an increased risk of kidney damage compared to their non-obese peers, characterized by a progressive decrease in kidney function and the development of proteinuria [15]. In adults, obesity-related glomerulopathy often presents as secondary focal segmental glomerulosclerosis, a histological pattern of kidney injury with specific histologic characteristics (e.g., perihilar sclerosis, segmental rather than global foot process effacement) resulting from podocyte damage and maladaptive hyperfiltration [16, 17]. Several intertwined factors contribute to kidney damage, including hyperfiltration leading to proteinuria and adipokines driving oxidative stress and inflammation, insulin resistance, and activation of the renin–angiotensin–aldosterone system (RAAS) alongside hypertension [18].

### Hyperfiltration and proteinuria

Glomerular hyperfiltration, characterized by a glomerular filtration rate (GFR) exceeding physiological levels, is an early feature of obesity-related kidney damage. This condition is closely associated with increased fat mass, which heightens metabolic demands and cardiac output, leading to increased kidney blood flow [19]. Other mechanisms, such as tubular sodium reabsorption and RAAS activation, triggered by decreased sodium delivery to the *macula densa*, contribute to afferent arteriole dilation, further promoting hyperfiltration [20]. Sustained hyperfiltration can compromise the integrity of the glomerular filtration barrier. In fact, increased kidney blood flow stretches the capillaries and expands the exchange surface, and in response, podocytes flatten their foot processes to try and maintain filtration. Despite this adaptive mechanism, the barrier's selectivity diminishes, resulting in microalbuminuria, the earliest detectable sign of kidney damage.

### Adipokines and inflammation

Adipose tissue secretes adipokines, small hormones that regulate various aspects of metabolism and inflammation. In obesity, the increased volume of adipose tissue leads to elevated levels of pro-inflammatory adipokines and a relative decrease in anti-inflammatory molecules, creating a state of chronic, low-grade inflammation. Leptin is a key regulator of satiety and exerts a direct effect on the kidneys. Elevated leptin levels and leptin resistance, defined as reduced sensitivity to leptin despite high blood levels, are commonly found in obese patients [21]. Leptin binds to its specific receptors on glomerular endothelial cells, activating inflammatory pathways, leading to TGF-β synthesis and driving mesangial hypertrophy, as well as extracellular matrix deposition within the glomerulus [22]. As a result, obese patients are at increased risk of developing proteinuria and eventually focal glomerulosclerosis [21]. Moreover, leptin induces oxidative stress in kidney tubular epithelial cells and activates monocytes to secrete IL-6 and TNF-α, further amplifying kidney inflammation [23]. TNF-α is a pro-inflammatory cytokine that might contribute to insulin resistance and endothelial dysfunction and directly damage the nephron due to the ability to stimulate the infiltration of inflammatory cells and activation of pro-inflammatory intracellular signaling pathways in kidney cells. IL-6 has a crucial role in systemic inflammation and exerts a direct impact on kidney cells, heightening inflammation and favoring extracellular matrix deposition [24]. Leptin might thus be a decisive central factor in the development of obesity-related kidney diseases [25]. In contrast to leptin, adiponectin is an anti-inflammatory adipokine that plays a protective role in metabolic and cardiovascular health. Adiponectin levels are typically reduced in obesity, and this deficiency has been associated with the development of proteinuria and glomerulosclerosis [26]. In animal models, adiponectin deficiency has been shown to increase podocyte injury and promote kidney inflammation and fibrosis, suggesting a pivotal role in the pathogenesis of obesity-related glomerulopathy [27, 28].

### Insulin resistance and type 2 diabetes mellitus

The pro-inflammatory state may also contribute to insulin resistance, which, along with hyperinsulinemia, is recognized as an independent risk factor for kidney injury [29]. These conditions have been shown to increase kidney blood flow and glomerular filtration rate [30], and they are linked to the rise in netrin-1, a biomarker demonstrating early tubular damage [31]. Type 2 diabetes mellitus, a common comorbidity of obesity, is often characterized by the development of diabetic nephropathy, marked by glomerular hyperfiltration, thickening of the glomerular basement membrane, and mesangial matrix expansion. These alterations result in microalbuminuria, proteinuria, and hypertension, which eventually lead to glomerulosclerosis and kidney failure. The onset of diabetic nephropathy in obese children and adolescents is becoming increasingly common, raising serious concerns [32].

### Hypertension and renin–angiotensin–aldosterone system

**Fig. 1:**
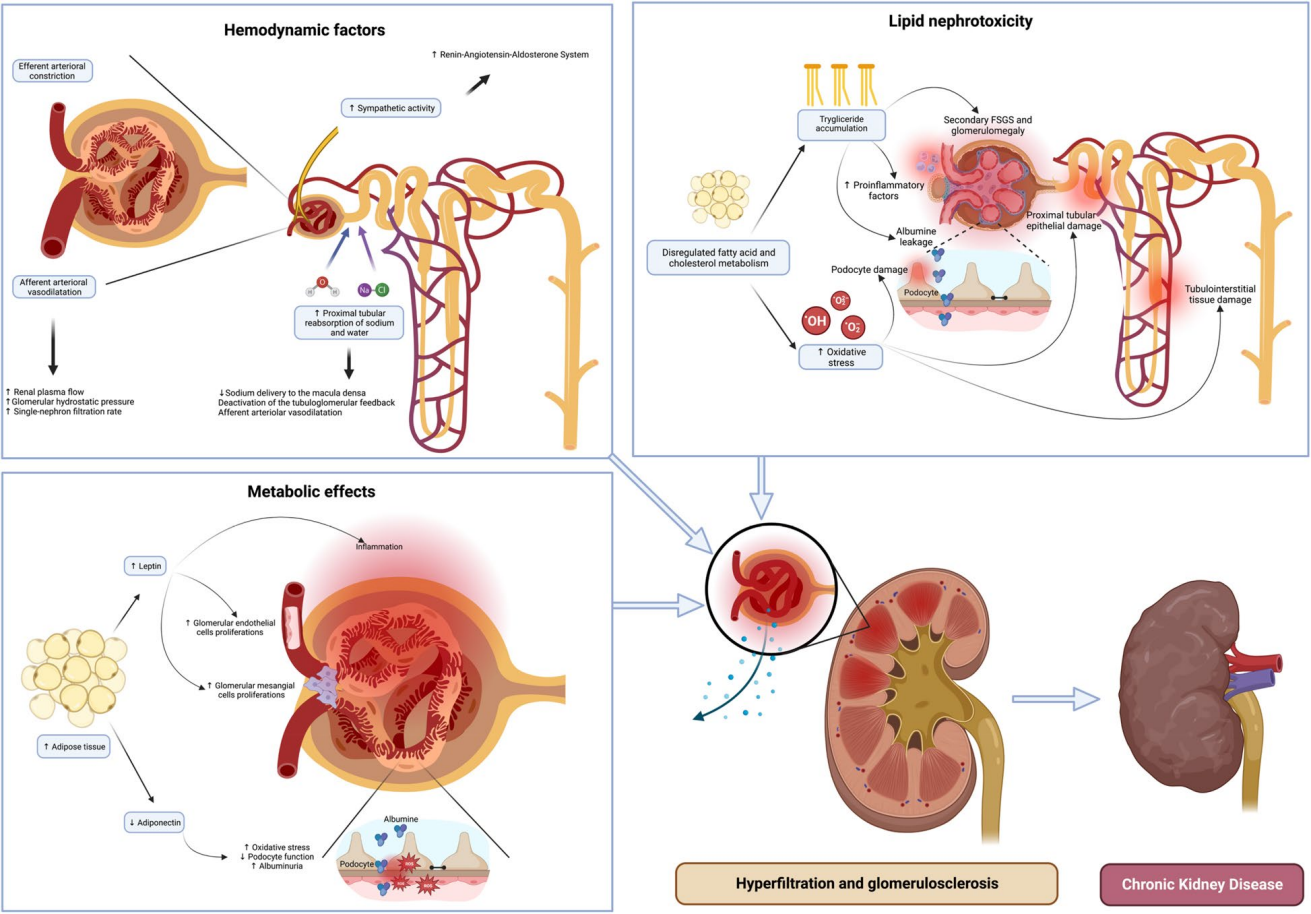
Pathophysiology of obesity-related kidney dysfunction. This figure depicts the critical pathways leading to kidney impairment in obesity, including hemodynamic alterations, metabolic disruptions, and lipid nephrotoxicity. These factors collectively contribute to kidney hyperfiltration and glomerulosclerosis, culminating in chronic kidney disease

BMI percentiles have been shown to closely correlate with blood pressure percentiles in children and adolescents, and fat mass seems to play a critical role in influencing hypertension [33]. Hypertension in obesity results from a complex interplay of factors, including the hyperactivation of the autonomic adrenergic system and the RAAS [34]. The tubuloglomerular feedback can regulate glomerular blood flow within normal pressure fluctuations, protecting the glomerular tuft. However, hypertension overwhelms this mechanism, exposing the glomeruli to damaging high pressure, leading to structural and functional damage, including endothelial injury, mesangial expansion, and podocyte stress. Over time, these changes impair the glomerular filtration barrier, contributing to proteinuria and accelerating the progression to glomerulosclerosis and chronic kidney disease (CKD) [35] (Fig. 1).

### Lipid nephrotoxicity and oxidative stress

Lipid nephrotoxicity is increasingly recognized as a significant contributor to obesity-related kidney injury. In obese individuals, excessive triglyceride accumulation within kidney tubular epithelial cells can lead to lipotoxicity, oxidative stress, and mitochondrial dysfunction. Lipid-mediated injury promotes inflammation, tubular cells apoptosis, and interstitial fibrosis, ultimately contributing to CKD progression. Lipid peroxidation and reactive oxygen species generation may represent final common metabolic pathways, contributing to and enhancing the development of obesity-related glomerulopathy [36, 37].

## Ultrasound applications

### B-mode

The use of US in the management of obese children can be considered for assessing morphological changes indicative of deteriorating kidney function, although functional assessment in children presenting with obesity-related hypertension is often more informative [38]. Nonetheless, the assessment of morphological parameters, including kidney length, width, depth, cortico-medullary thickness, echogenicity, and volume, provides important insights into kidney health and function.

**Fig. 2:**
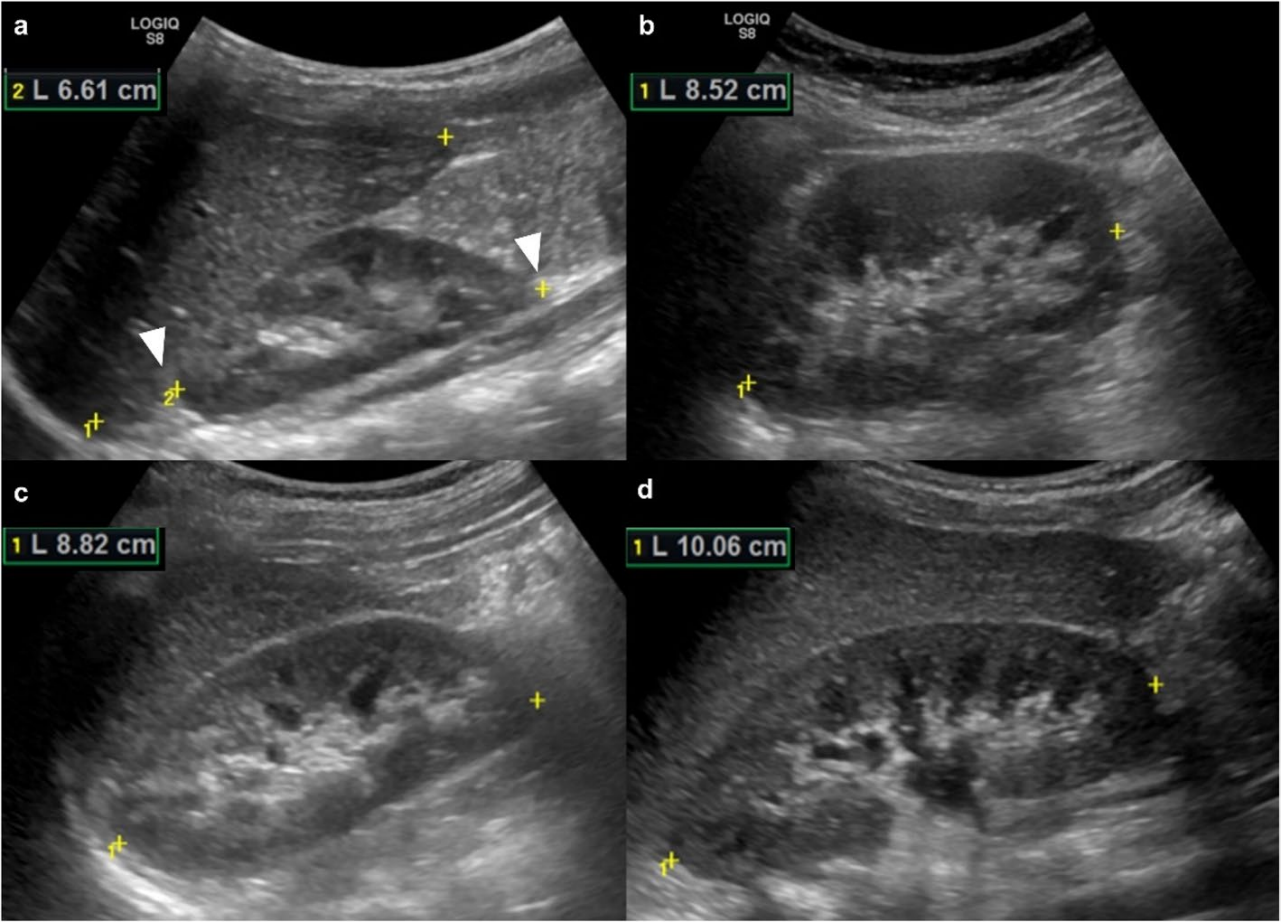
Kidney length. B-mode US images of the right kidney on the longitudinal scan plane, respectively in 2-year-old (**a**), 8-year-old (**b**), 11-year-old (**c**), and 17-year-old (**d**) patients, allow us to measure the craniocaudal kidney length. The measurement calipers are set (white arrowheads), showing different measures, respectively 6 cm (**a**) in the toddler, 8 cm in the school-aged children (**b**,**c**), and 10 cm (**d**) in the adolescent patient

Kidney size was proved to correlate well curvilinearly to age while linearly to body height, body weight, and body mass index [39]. A size reduction indicates advanced kidney damage, with sclerosis following prolonged exposure to the described pathophysiological processes [40]. Kidney length is defined as the maximum distance from the superior to the inferior pole, measured in a longitudinal view (Fig. 2).

**Fig. 3:**
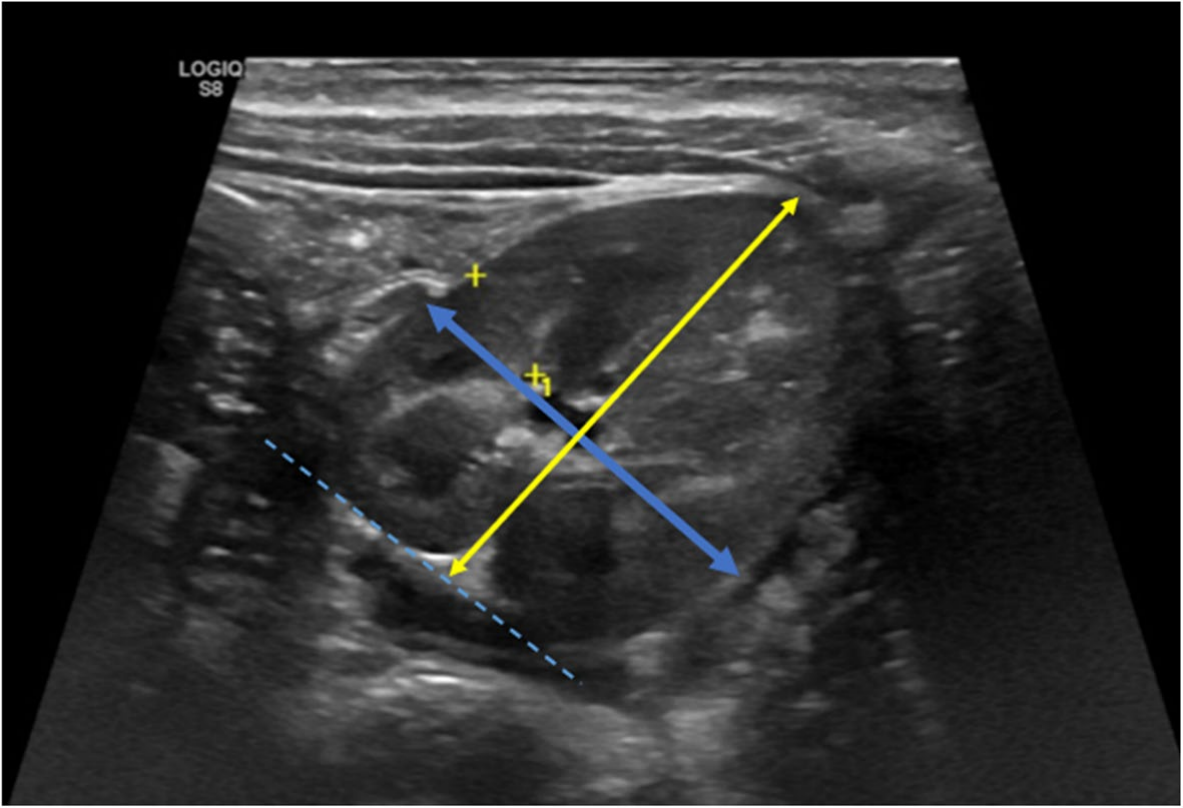
Kidney width and depth. B-mode US image of the kidney on the transversal scan plane (short axis), in a 2-year-old patient, allows us to measure respectively the width (yellow arrow) and depth (blue arrow)

Normal kidney length ranges from approximately 4.5–6.2 cm in neonates/toddlers (0–12 months), 6.6–8.3 cm (1–8 y) in pre/grade-schoolers, 8.9–10.4 cm (8–13 y) in grade-schoolers children, and 9.8–10.8 cm in (13–18 y) teens [41]. In addition to age, kidney length in children is affected by several other factors, including gender, height, weight, and ethnicity [42, 43]. It has been found that measurement of kidney length was independently associated with BMI, and a kidney length nomogram for obese children was proposed (kidney length (mm) = 62.79 + (1.36 × BMI)) (R2 = 0.32; p = 0.0001) [43]. Regarding the kidney width, it is measured on a transverse scan, and the maximum transverse diameter at the hilum is taken as the kidney width (Fig. 3).

**Fig. 4:**
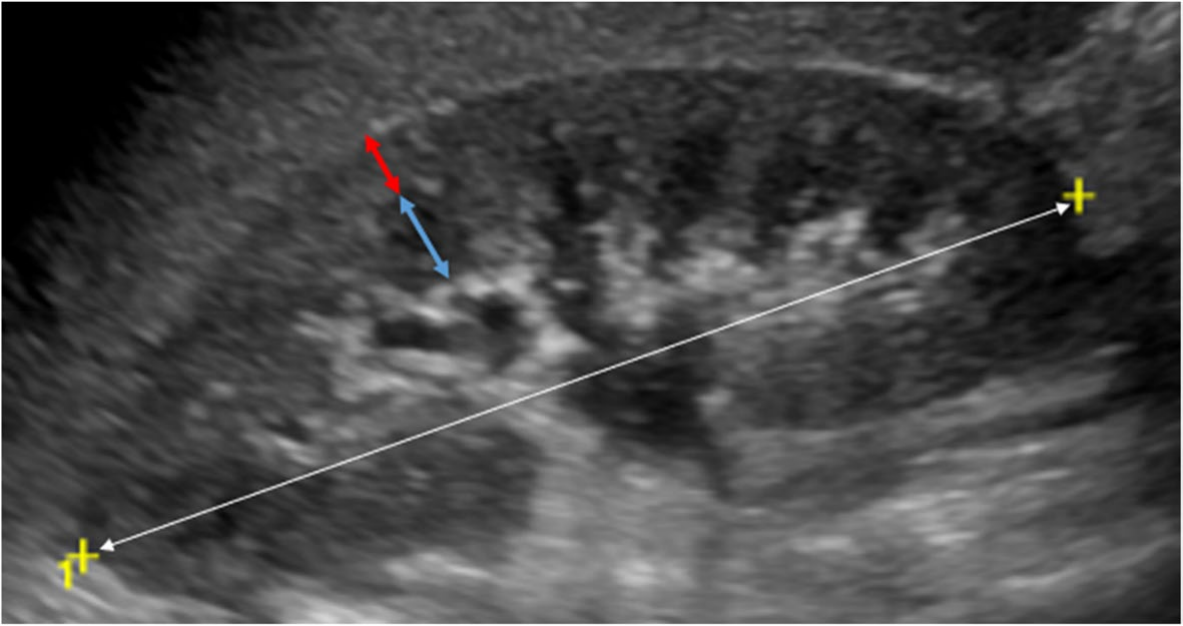
Kidney cortical thickness. B-mode US image of the right kidney on the longitudinal scan plane (white arrow, long axis), in a 17-year-old patient, allows us to measure respectively the cortical thickness (red arrow), measured perpendicularly from the outer margin of the kidney to the corticomedullary junction, and medullary thickness (blue arrow)

These measurements allow US-based kidney volume calculation using the ellipsoid formula (length x width x depth × 0.523), considered less reliable than MRI evaluation, due to the risk of underestimation of true kidney volume by 21–29% [44]. Volumetric analysis is critical in identifying pathological hypertrophy, particularly in obese patients. Kidney volume varies significantly with age, measuring approximately 15–28 cm^3^ (0–6 months) in neonates/toddlers, 31–76 cm^3^ (1–8 y) in pre/grade-schoolers, 76–108 cm^3^ (8-13y) in grade-schoolers and 108–200 cm^3^ (13-18y) in teens [45, 46]. Finally, the kidney cortical thickness is defined as the maximal length from the fat interface to the cortex-pyramidal base interface measured perpendicularly under longitudinal US (Fig. 4).

Kidney cortical thickness below 6 mm may indicate cortical atrophy, often the expression of CKD secondary to the condition [47].

Moreover, if US-determined kidney volume was found to be correlated with GFR in CKD adult patients also a statistically significant positive association was found between estimated GFR and mean kidney length (r = 0.66, *p* < 0.01) and between estimated GFR and mean cortical thickness (r = 0.85, *p* < 0.01), with the latter being more prominent [47, 48]. Cortical thickness measured on ultrasound appears to be more closely related to GFR than kidney length, and reporting cortical thickness should always be considered in patients with CKD [49].

Kidney echogenicity is an important parameter in US examinations. In adults, it is compared to the echogenicity of the liver or spleen to identify variations from normal parenchymal patterns. Increased echogenicity may reflect changes in the kidney parenchyma, ranging from benign processes to pathological conditions such as obesity-related glomerulopathy. Kidneys with higher echogenicity than the liver or spleen may harbor sclerosis or fat content, both common in obese patients [9]. Moreover, differences exist in the kidneys of neonates compared to those of older children and adults. Neonates have a lower content of sinus fat, which makes their central kidney echogenicity less prominent. Additionally, the kidney cortex in neonates is typically hyperechoic when compared to the liver. This hyperechoic nature gradually diminishes by the age of 4 to 6 months, eventually leading to the cortex becoming hypoechoic. In healthy children, except for transient hyperechogenic medullary pyramids in neonates, kidney pyramids appear hypoechoic in comparison to the kidney cortex, due to the cortico-medullary differentiation [50]. Furthermore, US measurement of perirenal fat thickness has been proposed to assess cardiovascular and renal risks, although data in children remain limited; it has been associated with early vascular changes that may contribute to hyperfiltration injury [51, 52]. Alternative screening tools for detecting early kidney damage in obese children have been investigated, and several urinary and blood biomarkers have been identified. Urinary N-acetyl-β-D-glucosaminidase (NAG) and kidney injury molecule-1 (KIM-1) levels have been found to be significantly higher in obese children compared to healthy controls, suggesting their utility in identifying early tubular damage [53]. Similarly, microalbuminuria and serum cystatin C have been proposed as early markers of glomerular injury in obese children [54]. Urinary netrin-1 and podocalyxin have also been reported as promising biomarkers for early tubular and glomerular damage in obese pediatric patients [31, 55].

Kidney US is then highly operator‑dependent; accuracy varies with sonographer experience, equipment quality, and patient body habitus. In obese children, increased subcutaneous fat and deeper organ depth reduce resolution and acoustic penetration, leading to lower reproducibility of measurements (e.g., cortical thickness, RI) and potential underestimation of parenchymal changes.

### Color-doppler

**Fig. 5:**
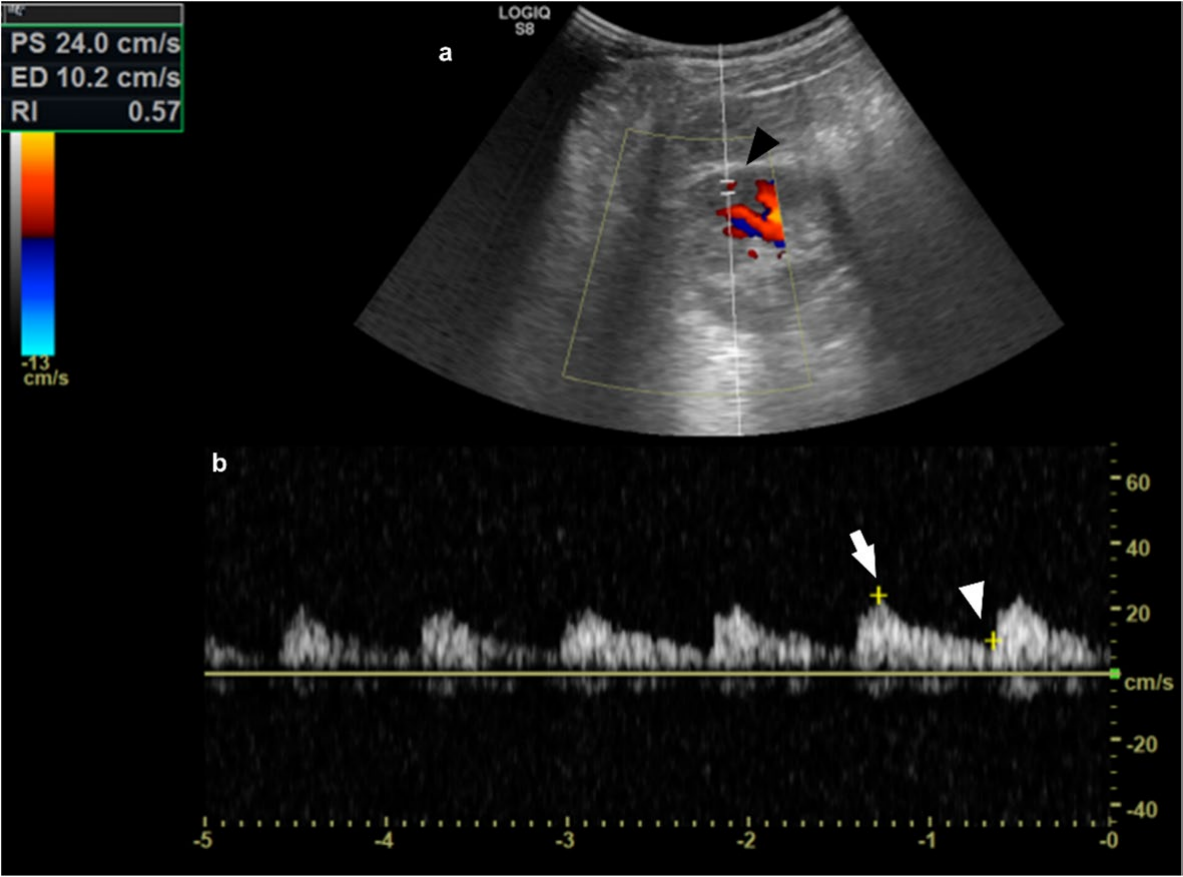
Kidney arterial RI measurement. (**a**) A sample volume (black arrowhead) is placed within an arcuate artery of an 11-year-old male patient, under color Doppler guidance, and spectral analysis of vascular signals (**b**) is obtained. The measurement calipers are set at the PSV (24.0 cm/sec, white arrow) and EDV (10.2 cm/sec, white arrowhead) of a waveform, and the RI is calculated (0.57) according to the formula (PSV-EDV)/PSV. EDV, end-diastolic velocity; RI, resistive index; SV, peak systolic velocity

Color Doppler US has become an important tool for the evaluation and assessment of various kidney diseases in children. Its advantages include being non-invasive and low cost, along with the capability to provide both anatomical details and real-time hemodynamic information about kidney perfusion [56]. The RI is the ratio between peak systolic and end diastolic velocity, which are derived from the Doppler spectrum of arcuate arteries, at the cortico-medullary junction, or interlobar arteries, adjacent to medullary pyramids (Fig. 5). The normal RI range in the adult population is 0.50–0.70, and higher values indicate increased kidney vascular resistance, often associated with a poorer prognosis in various kidney diseases. Numerous studies have suggested that the mean kidney RI is dependent on age, showing an overall decreasing trend as age increases. The RI is typically highest at birth, often exceeding the normal upper limit of 0.70 during the first year of life, particularly during the first six months [57, 58]. As children grow, the RI gradually declines, reaching adult levels between the ages of 4 and 7 years. An increased RI reflects early vascular damage in patients with CKD and hypertension, as it may reflect changes in kidney microcirculation, including glomerular hyperfiltration (42,43). Numerous studies confirm RI effectiveness in monitoring acute kidney injuries in pediatric intensive care setting [59, 60], and a growing literature is confirming its utility and reliability in a vast range of pediatric CKD, including kidney assessment in pediatric hypertension and type 1 diabetes mellitus [61, 62]. While its use in the adult population with severe obesity has been studied [63], RI role as an early indicator of vascular damage in obesity-related glomerulopathy in children warrants targeted studies on this population, as current evidence is inconclusive.

Pediatric hypertension guidelines and AAP recommend Doppler kidney US as a non-invasive screening tool for evaluating renal artery stenosis in non-obese children and adolescents aged 8 years and older who are suspected of having renovascular hypertension [64], highlighting a limited application in obese children due to technical and vascular biases that compromise its accuracy [14].

### Emerging US technologies

Technological innovation has introduced new diagnostic tools that hold significant potential for future use, with other methods such as CEUS and elastography. These techniques enable precise evaluation of kidney perfusion and tissue stiffness, offering valuable insights into early pathological changes linked to obesity-related kidney damage and improving prognostic assessments [65]. CEUS is an innovative imaging technique that utilizes biologically inert microbubbles, which consist of a gas core stabilized by a lipid monolayer shell with a diameter of less than 10 µm. This US contrast agent is approved by the US Food and Drug Administration for intravesical use in evaluating vesicoureteral reflux in children. However, it is still considered off-label for kidney applications [66]. CEUS has demonstrated greater accuracy in detecting perfusion abnormalities in the kidney microvasculature compared to Doppler US. This improved performance is attributed to the nature of the contrast agent, purely intravascular. On the other hand, Doppler US relies on detecting frequency shifts, that is significantly influenced by acquisition speed, imaging angle, depth, and frequency [67]. This technique can improve the visualization of perfusion, providing quantitative values through the observation of time-intensity curves: time to peak, rising time, area under the curve, peak intensity, and mean transit time [68–71].

Another advanced US technique is elastography, a type of US imaging used to measure tissue elasticity through the transmission of acoustic energy into the body to cause transient displacements of the tissue. The US transducer detects deformations because the displacement is slower than the speed of the US pulse waves. These deformations determine the tissue’s elasticity, which can be measured in either a relative or absolute manner, generally expressed in kPa. Considering fibrosis, a hallmark of CKD, there is an increase in collagen deposition and extracellular matrix at the microscopic level, impacting the overall stiffness of the kidney. It has been observed that lower elasticity values in patients with advanced CKD, with shear wave elastography that seems to be superior to kidney length and cortical thickness as the US markers of CKD, highlighting a cutoff value for healthy vs diseased native kidneys of 4.31 kPa [72]. Despite these results, there remains considerable variability in kidney elastography findings in the literature. This variability can be attributed to several factors that may affect this technique, including blood and urinary pressures, the orientation of the excitation source, hydration status, glomerular hypertrophy and sclerosis, as well as cardiac and respiratory cycles [73–75]. Table 1 provides a summary of our suggested practice recommendations for early kidney damage screening in obese children and adolescents.

**Table 1:** Key practice recommendations

In obese pediatric patients with cardiovascular risk factors, perform baseline ultrasound (B-mode + Doppler) together with urinary microalbumin/creatinine ratio and serum cystatin C
Use cortical thickness and resistive index measurements for longitudinal monitoring; consider perirenal fat thickness as a supplementary risk metric
Repeat screening every 12–18 months, or sooner if clinical or laboratory abnormalities develop
Collaborate with pediatric nephrologists, endocrinologists, radiologists and dietitians to address modifiable risk factors and interpret imaging/biomarkers

## Conclusion

US is a non-invasive and accessible imaging modality, effective in detecting morphological and functional changes in kidney diseases. Due to the limited evidence in the literature, its use as a primary tool for the early detection of structural and vascular damage, specifically related to obesity-associated glomerulopathy, is not yet supported. Indeed, in early stages, US findings are often normal, and typical morphologic signs of kidney injury, such as increased echogenicity and loss of corticomedullary differentiation, generally appear only in advanced disease. On the other hand, the functional parameter RI is showing promising results in detecting early vascular changes in acute kidney injury in pediatric intensive care setting, and in other CKD. The utility of RI in assessing obesity-related glomerulopathy in children has not been conclusively demonstrated, and dedicated studies in this population are still needed. Similarly, emerging technologies like CEUS and kidney elastography appear promising for monitoring vascular damage and kidney sclerosis. Evidence supporting their applicability in pediatric patients is currently lacking. Therefore, screening strategies for early renal injury in this population should consider incorporating urinary and serum biomarkers. New and emerging markers have shown promise in identifying early tubular and glomerular damage. In conclusion, the US could be considered a strategic adjunct to comprehensive clinical evaluation and multimodal diagnostics, driving proactive prevention and personalized treatment strategies in obese pediatric patients.

## Data Availability

The datasets generated during and/or analyzed during the current study are available from the corresponding author upon reasonable request.

## Abbreviations

US: Ultrasonography/ultrasounds
RI: Resistive index
CEUS: contrast-enhanced ultrasonography
RAAS: Renin–angiotensin–aldosterone system
GFR: Glomerular filtration rate
CKD: Chronic kidney disease
